# Unraveling Anatomical Variations of the Acromioclavicular Joint: A Comprehensive Inquiry Into Their Impact on Instability

**DOI:** 10.7759/cureus.75197

**Published:** 2024-12-06

**Authors:** Charikleia M Skarpeti, Stavros Angelis, Alexandros Apostolopoulos, Dimitrios Filippou

**Affiliations:** 1 Anatomy, National and Kapodistrian University of Athens, Athens, GRC

**Keywords:** acromioclavicular joint, acromion, anatomical variations, instability, shoulder biomechanics

## Abstract

The acromioclavicular (AC) joint, an essential element in the complex biomechanics of the shoulder, displays a diverse range of anatomical variations among individuals. This review aimed to study and present these variations. A detailed search was conducted on the PubMed medical database by using the terms “acromioclavicular joint variations”. Initially, 108 articles were retrieved and, after applying the inclusion and exclusion criteria, only 11 articles were found eligible. Eight more papers were selected from the citations in those 108 papers. As a result, a total of 19 papers and studies were used for the final analysis.

This comprehensive review highlights the existence of a wide range of anatomical variations, concerning joint alignment, dimensions of articular surfaces, ligament arrangements, as well as acromion types and joint angles. Gender-related differences in joint dimensions and angles, as well as comparisons between intact groups and patients with rotator cuff tears, are also examined. Delving into the intricacies of anatomical variations is essential in comprehending how these differences may contribute to joint instability. Through a synthesis of anatomical studies, clinical observations, and biomechanical analyses, this narrative review provides an up-to-date assessment of the existing information regarding the AC joint.

## Introduction and background

The acromioclavicular (AC) joint constitutes a critical component of the shoulder complex, serving as a pivotal linkage between the thorax and the upper limb. Its diverse anatomical forms have sparked interest among medical professionals, scholars, and anatomists, due to their potential implications on the instability and function of the joint [1,2]. Understanding the anatomical intricacies of the AC joint is crucial for both clinicians and researchers, as it underpins accurate diagnosis, treatment planning, and surgical decision-making in individuals with shoulder pathology. A thorough review of the current body of knowledge will be essential to integrate various research findings and confirm the presence of consistent anatomical patterns. The purpose of this study is to provide a comprehensive overview of the joint’s alternative anatomy and to highlight how this could affect stability.

## Review

Materials and methods

An advanced search was conducted on PubMed using the term "acromioclavicular joint variations." The initial search involved the examination of titles, leading to the screening of abstracts for relevance. Only English-language studies involving human subjects that examined AC joint variations were included, whereas studies that involved non-human subjects, those in languages other than English, or those that lacked a specific focus on AC joint variations were excluded. Data extracted included the number and type of patients examined, the methods of examination, categorization of variations, and study results. Subsequently, additional references were sourced by examining citations from the initial papers.

Results

**Figure 1:**
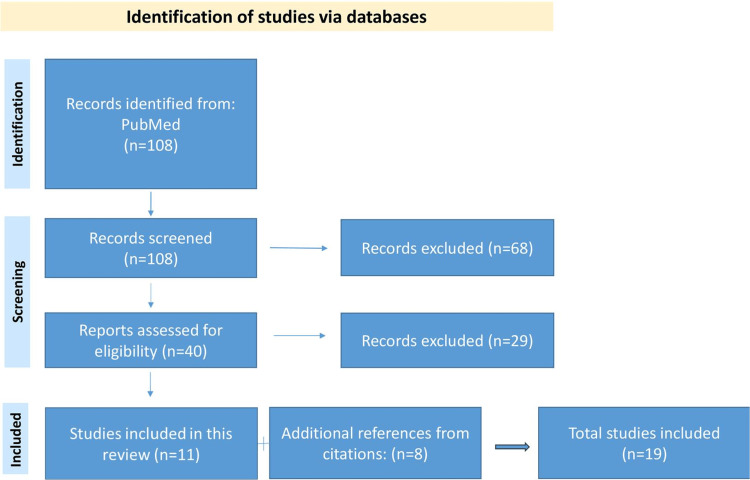
Flowchart illustrating the screening process

The initial search on PubMed yielded 108 papers. After title screening, 68 papers were eliminated, leaving 40 papers for abstract screening. Out of these, only 11 met the inclusion criteria, while 29 were excluded due to irrelevance. A secondary search of citations of the initial 108 papers led to the inclusion of eight additional papers; hence, 19 papers were included in the final analysis. The flow chart of the screening process is depicted in Figure 1.

Discussion

Basic Anatomical Considerations

It is well known that the AC joint is the articulation between the acromion of the scapula and the distal end of the clavicle. It is a diarthrodial joint, allowing free movement, and a part of the pectoral girdle. It is classified as a synovial joint, particularly a plane joint, which indicates the presence of a joint cavity and the allowance of smooth, gliding movements. The articular surfaces of the bones involved are covered with fibrocartilage, which is well suited for this specific area subjected to tensile and compressive forces.

The AC joint is enveloped by a fibrous joint capsule, offering stability and containing synovial fluid. This fluid lubricates the joint, minimizing friction and enabling seamless movement. The joint capsule consists of a synovial membrane, which secretes fluid, and an outer fibrous membrane for support. Within the AC joint, there is an intra-articular cartilaginous disk that enhances its overall stability and function [2]. The AC joint serves as a linkage between the thorax and the scapula, thereby enhancing the range of motion of the latter and simultaneously aiding in transmitting forces from the upper arm to the rest of the skeletal structure. In addition, it assists arm movements, such as the flexion and the abduction of the shoulder. Its dynamic function underscores its significance in orchestrating coordinated movements essential for optimal upper limb mobility and functionality [3].

Variations

Anatomical variations can be seen in the AC joint and may involve differences in the joint components' shape, size, or alignment. Although some variations can be a part of natural heterogeneity, several can be associated with the augmentation of risk for conditions such as shoulder instability.

Articulation patterns: The AC Joint exhibits a wide range of variations in its articulation patterns. Urist executed 100 roentgenograms, highlighting the alternative articulation patterns [4]. In 49% of cases, the articular surface of the clavicle was found to override that of the acromion, whereas the inferior margin of the clavicle's articular surface under-riding the superior margin of the acromion was only observed in 3% of cases. Additionally, 27% of cases exhibited nearly vertical and co-planar articulation surfaces for both the acromion and clavicle. The rest of the cases presented incongruent surfaces where the acromion process did not align with the clavicle. Moreover, meniscus types varied, from a fibrocartilage blade to a complete disc. Some cases lacked a distinct diarthrosis, represented only by a bundle of fibrous tissue.

In a complementary study, Colegate-Stone et al. conducted cadaveric and radiological assessments, as well as CT analysis of AC joints, which were classified based on their morphology revealing the three following 3D types: flat, oblique, and curved [5]. Based on the statistical analysis (Student's t-test) there was no significant difference in the frequency of flat, oblique, or curved joints among cadaveric examination, CT analysis, and radiological appearance. Edelson examined 280 skeletons and measured the obliquity of the acromial facet from the vertical using a goniometer [6].

Despite variability in the AC joint configuration, a consistent pattern of degenerative changes was observed. Elongation in the sagittal plane, primarily in the posterior aspect of the acromial facet, occurred in 73% of cases, with 64% involving the right side when unilateral. Anteroposterior elongation often coincided with less prominent osteophyte proliferation in the superoinferior plane, especially at the posterosuperior quadrant of the acromial facet. On the side of the clavicle, the distal end underwent a widening and rounding process in the front-to-back direction, leading to a modified ball-and-socket shape. Minor variations existed based on the initial AC joint anatomy, with only 18% of specimens having a facet inclination of 30° or more from the vertical. Arthritic changes in those oblique joints were similar to more vertical specimens. Still, differences included a more prominent inferior lip of the acromial facet and a more rounded-off inferior aspect of the clavicle.

Renfree and Wright in their review mention that the most prevalent joint configuration involves the clavicle over-riding the acromion while the least common is when it underrides the acromion [7]. The angles of inclination may vary since the clavicle can override the acromion by up to 50 degrees. While some studies have found no correlation between the inclination of the AC joint and arthritis, other researchers suggest that joints with a more vertical orientation may be more prone to osteolysis due to increased forces concentrated at the distal clavicle. On average, the size of joint surfaces in the adult acromioclavicular joint is around 9 mm vertically and 19 mm anteroposteriorly. The width of the AC joint typically ranges from 1 mm to 3 mm, decreasing with age. Although an articular disc is typically present within the joint, its size, shape, and completeness can vary among individuals.

Angles and alignment: Variations can also occur when it comes to the angles and the alignment between the joint’s components. Kim et al. performed 101 CT scans, revealing a mean angle between the distal end of the clavicle and the acromion to be 17.1° ± 10.51° and an average height difference of the same anatomical structures to be 3.5 ± 1.65 mm [8]. Concurrently, Helleberg et al. executed a total of 204 MRI scans which indicated that the axial plane exhibited the highest visibility at 84.8%, compared to the coronal and the sagittal plane [9]. In addition to this, the study revealed the average angles between the AC joint and the glenoid to be 41.1° in the axial, 34.4° in the coronal, and 52.5°in the sagittal planes.

Nakazawa et al. discerned two distinctive segments within the AC ligament, a superoposterior (SP) bundle, that exhibited consistent and robust development, running obliquely from the superior surface of the anterior acromion to the posterior part of the distal clavicle and an anteroinferior (AI) bundle, situated on the anteroinferior side of the joint, which was characterized as thin and narrow [10]. The study quantified the angle between the SP bundle and the perpendicular line to the joint surface, using a digital goniometer, yielding an average of 30° (±6°), with a range from 13.8° to 38.6°. Furthermore, the angle measured between the sagittal plane of the joint surface and the long axis of the distal clavicle averaged 70° (±12°), ranging from 34.9° to 85.9°. Notably, a moderate negative correlation was identified between joint inclination and the angle of the ligament, indicating that as joint inclination increased, the ligament angle tended to decrease.

The investigation revealed diverse configurations in the AI bundle leading to its classification into three distinct types based on structural variations. Approximately 42.3% of the specimens displayed an AI bundle extending from the anterior surface of the acromion to the anterior edge of the distal clavicle (type 1). In an equal percentage, the AI bundle reached from the anterior to the inferior surface of the joint capsule but did not entirely cover the inferior joint surface (type 2). The remaining 15.4% exhibited an AI bundle extending from the anterior to the superior surface of the joint capsule (type 3). In contrast, the SP bundle exhibited consistent and uniform characteristics.

Gender-based variations: It is important to highlight that variations in the configuration and angles of the joint can also occur between the two genders. Gender-based comparisons revealed significant anatomical differences. Females exhibited a notably higher mean AC angle, whereas males had significantly greater mean depth of the acromion and AC height difference, as well as thicker acromion and distal clavicle measurements, highlighting discernible variations in shoulder anatomy between genders [8].

Joint biomechanics: Variations can also occur concerning the joint’s biomechanics, the disruption of which can lead to significant multidirectional instability. Flores et al. in their study investigated the biomechanical role of the acromioclavicular ligament by testing cadaveric shoulders on a specialized platform to measure force-displacement in different directions before and after cutting key ligaments, including the acromioclavicular ligament [11]. Using the data, computer models were created to simulate ligament behavior, revealing that the acromioclavicular ligament had an average free length of 11.0 mm and a stiffness of 33.8 N/mm. The accuracy of these models was confirmed by the close match between predicted and experimental results.

The acromion: The acromion process of the scapula itself presents an array of variations, which are of significant interest in understanding shoulder joint biomechanics. Kim et al. observed moderate variability in the depth and thickness of the acromion, as well as the distal clavicle [8]. The average acromion thickness measured 8.3 ± 1.07 mm, while the distal clavicle thickness averaged 11.7 ± 1.76 mm. The Bigliani classification system, proposed in 1986, introduced three types of acromion: Type I (flat), Type II (curved), and Type III (hooked), with Type IV (convex) later added. Notably, multiple studies have consistently linked Type III acromions with an increased likelihood of rotator cuff tears [12].

A study of 210 specimens (420 scapulas) from the Hamann-Todd Osteological Collection at the Cleveland Museum of Natural History examined the influence of age on acromial morphology [13]. The sample included equal numbers of male and female, black and white subjects. Researchers measured the acromion's length, width, and anterior thickness, along with the acromial facet of the acromioclavicular joint, using digital calipers and radiographic evaluations. Acromial morphologies were categorized as type I (flat) in 32%, type II (curved) in 42%, and type III (hooked) in 26% of cases. The study found no consistent, statistically significant impact of age on these morphologic conditions. Os acromiale was present in 8% of specimens, with 41% showing bilateral involvement. Men had larger mean acromial dimensions than women. Multiple regression analysis showed no significant age-related changes in these dimensions. However, a significant age-related increase in degenerative changes was observed, with anterior acromial spur formation increasing from 7% in those under 50 to 30% in those over 50 (p<0.05). The study concluded that acromial morphology is primarily an anatomical characteristic independent of age and contributes to impingement disease alongside age-related degenerative changes.

According to Prescher the acromion typically forms through the fusion of multiple ossification centers [1]. This process is typically finished by the age of 25. There are three main elements: the preacromion, mesacromion, and metacromion, which merge to form a triangular epiphyseal bone, eventually fusing with the basiacromion. However, in a minority of cases (around 7-15%), disruptions in this fusion process can lead to a condition known as 'os acromiale'. The diarthrosis of the acromial bone and the basiacromion is referred to as the interacromial articulation. In cases of os acromiale, the clavicular facet of the acromion may be situated entirely on the os acromiale or divided between the basiacromion and the os acromiale. In the first scenario, movements of the shoulder girdle place significant strain on the interacromial articulation, leading to premature osteoarthritis.

AC joint duplication: Lastly, there is an extremely rare variation called AC joint duplication, with only seven cases reported since 1915. This condition, sometimes referred to as "bifid clavicle" or "clavicular bifurcation," has been linked to potential genetic factors or trauma during growth. Unlike more common coracoclavicular joints or calcifications of coracoclavicular ligaments, true acromioclavicular duplications have been rarely documented. Viard et al. presented a unique case of a 51-year-old patient with a complete left acromioclavicular joint duplication, who experienced non-traumatic shoulder pain and disability [14]. For the first time, three-dimensional CT-scan reconstructions provided new insights into this rare morphological variation. Previous reports of clavicular duplication have often been post-mortem or incidental, with limited clinical implications. This case, illustrated with advanced 3D imaging, is the first detailed account of such a duplication with clinical context, although the exact relationship between the duplication and the patient's symptoms remains uncertain.

Impact of rotator cuff tears on joint morphology: The appearance of the AC joint can vary significantly between healthy individuals and patients with rotator cuff tears, reflecting alterations in joint morphology, position, and space. Cuomo et al. conducted an anatomic study on 123 shoulders and compared groups that presented full-thickness rotator cuff tears (RCT) to intact groups [15]. No significant differences were observed in the AC joint position within the supraspinatus outlet in relation to the center of the glenoid reference within the two groups. On average, the intact group had the AC joint positioned 10 mm posterior and 4.5 mm lateral to the glenoid center, while the RCT group had it located 9.8 mm posterior and 5.5 mm lateral. Furthermore, the study involved measuring the distances between the humeral head and the AC joint, as well as the humeral head and the lower part of the anterolateral corner of the acromion to assess the maximum clearance space for the supraspinatus tendon within the outlet. The RCT group showed significantly less clearance, with 6.9 mm between the humeral head and AC joint (compared to 9.8 mm in the intact group) and 3.6 mm between the humeral head and the undersurface of the anterolateral corner of the acromion (compared to 6.9 mm in the intact group).

These results indicate diminished clearance space in the presence of rotator cuff tears. The study also assessed both joint space width and the presence and size of osteophytes within the two groups. The RCT group demonstrated a significantly narrower joint space (1.77 mm) compared to the intact group (2.41 mm) Calculated osteophyte sizes were notably larger on both sides of the acromioclavicular (AC) joint in the RCT group. Specifically, on the acromial side, RCT shoulders had 1.8 mm osteophytes, contrasting with the 0.3 mm in intact shoulders. On the clavicular side, the measurements were 2.8 mm in the RCT group and 1.8 mm in the intact group. Direct measurements of inferior acromial osteophytes were significantly larger in RCT shoulders (1.23 mm) than in intact shoulders (0.6 mm). Major osteophytes (≥2 mm) were present in 62.5% of RCT shoulders, a significantly higher incidence than the 12.5% observed in intact shoulders (P < .001). In summary, the RCT group exhibited considerable joint space narrowing and larger osteophytes, indicative of more pronounced degenerative changes in the AC joint.

Treatment options: Various treatment and surgical reconstruction strategies exist, but determining which treatment option offers the best stability remains unanswered. Beitzel et al. compared different surgical techniques for reconstructing acromioclavicular (AC) joint dislocations, focusing on both coracoclavicular (CC) and AC ligament reconstruction [16]. Using 24 cadaveric shoulders tested with a servohydraulic system, four reconstruction methods were evaluated for their impact on joint stability, measuring anterior, posterior, and superior translation, as well as rotation. The results showed that the technique involving direct wrapping and suturing of graft around the AC joint (group 1) provided the most stability, closely replicating the native joint's behavior, particularly in anterior rotation and minimizing posterior and superior translation. In contrast, the transacromial technique resulted in the most significant translation and rotation, deviating the most from native joint function. The study concluded that anatomic repair should involve both the CC and AC ligaments to maintain optimal physiological movement and stability of the AC joint.

However, Scillia and Cain described a different technique - anatomic coracoclavicular (CC) ligament reconstruction with suture augmentation-which is believed to offer stability comparable to the intact ligamentous state of the AC joint [17]. The use of autografts in this technique provides a dependable tissue structure for healing in both acute and chronic injuries. Additionally, the use of braided PDS sutures offers temporary fixation strength while avoiding the potential long-term complications associated with rigid nonabsorbable sutures or tapes. The surgical technique presented here modifies the anatomic CC ligament reconstruction to improve anterior-posterior stability, which is particularly crucial for performance in overhead athletes.

Moreover, Vereb et al. in another prospective study addressed the clinical need for alternative treatments in patients with persistent painful arthritis of the AC joint who had not responded to previous therapies [18]. It specifically evaluated the efficacy and safety of radiosynoviorthesis (RSO) using erbium-169 citrate in 51 patients with arthritis in 85 AC joints. The results showed a significant reduction in pain, with a mean decrease of 47% on the Visual Analogue Scale (VAS) at six months post-treatment. The global therapeutic effect was rated as excellent or good in 96% of the joints treated. Additionally, a significant correlation was found between the decrease in pain and the reduction in blood perfusion to the joint, as measured by the target/non-target uptake ratio (T/NTR) on bone scintigraphy. However, a specific cut-off value for T/NTR to predict therapeutic response could not be established. Overall, the study concluded that RSO with erbium-169 citrate is a safe and effective option for managing AC joint arthritis when other treatments have failed.

Polisetty et al. in their study, focused on identifying potential risk factors associated with acromion fractures following reverse shoulder arthroplasty (RSA), specifically examining the role of the AC joint and the positioning of the humerus and glenoid [19]. In a retrospective analysis of 920 RSA cases, 47 patients (5.1%) experienced postoperative acromion fractures, which were compared to a control group of 141 patients matched by age, gender, and surgical indication. Despite thorough analysis of preoperative and postoperative radiographs, including measurements of critical shoulder angle, acromion-humeral interval, global lateralization, and delta angle, as well as a computed tomography assessment of AC joint morphology, joint space, and osteoarthritis indicators, no significant differences were found between the fracture and nonfracture groups. The study concluded that there is no clear association between AC joint characteristics or humeral and glenoid positioning with the occurrence of postoperative acromion fractures after RSA.

This comprehensive review meticulously investigates the anatomical variations of the AC joint, unveiling a spectrum of intriguing findings. The observed diversity encompasses discrepancies in joint component alignment, variations in the size and shape of articular surfaces, and distinctive configurations of ligaments. In addition, variations associated with acromion types, joint inclination, the angle between the components, as well as their depth and thickness are observed. Several comparisons are included in this review, such as gender-based differences that indicate variations in joint angles and dimensions between males and females. Another comparison included differentiates intact groups with groups presented with rotator cuff tears, revealing variations associated with the presence or absence as well as the size of osteophytes and the width of the joint space.

Awareness of these variations is vital in the context of medical imaging and diagnostic procedures. Radiologists and clinicians interpreting imaging studies need to recognize and differentiate between normal variants and potential abnormalities to make accurate diagnoses and treatment plans. Understanding the individual variations is essential in orthopedics for implementing personalized surgical approaches, ensuring precision and effectiveness in patient care. Moreover, an understanding of the joint’s variations is relevant in the broader context of musculoskeletal health, since certain variations may be associated with an increased risk of specific conditions, such as shoulder instability or rotator cuff tears. This knowledge can guide preventive measures, and early interventions and may reduce the risk of associated complications.

## Conclusions

This comprehensive review examines the anatomical variations of the AC joint. The diversity explored includes differences in the configuration and morphology of joint components, and variations in the alignment and the angles of the joint. Gender-based disparities in joint angles and dimensions between males and females are also highlighted, alongside comparisons distinguishing intact groups from those with rotator cuff tears, which reveal variations in the width of the joint space and the presence or absence, as well as the size, of osteophytes. Variations are important as they signal the need for personalized medicine, where treatments and interventions can be tailored to individual anatomical differences, optimizing patient care and outcomes. In the case of the AC joint, being aware of variations in joint angles, dimensions, and ligament configurations can guide treatment strategies, influencing not only surgical approaches but also rehabilitation plans tailored to accommodate individual differences.

## References

[1] Prescher A (2000). Anatomical basics, variations, and degenerative changes of the shoulder joint and shoulder girdle. Eur J Radiol.

[2] Bearden JM, Hughston JC, Whatley GS (1973). Acromioclavicular dislocation: method of treatment. J Sports Med.

[3] Flores DV, Goes PK, Gómez CM, Umpire DF, Pathria MN (2020). Imaging of the acromioclavicular joint: anatomy, function, pathologic features, and treatment. Radiographics.

[4] Urist MR (1946). Complete dislocations of the acromioclavicular joint; the nature of the traumatic lesion and effective methods of treatment with an analysis of forty-one cases. J Bone Joint Surg Am.

[5] Colegate-Stone T, Allom R, Singh R, Elias DA, Standring S, Sinha J (2010). Classification of the morphology of the acromioclavicular joint using cadaveric and radiological analysis. J Bone Joint Surg Br.

[6] Edelson JG (1996). Patterns of degenerative change in the acromioclavicular joint. J Bone Joint Surg Br.

[7] Renfree KJ, Wright TW (2003). Anatomy and biomechanics of the acromioclavicular and sternoclavicular joints. Clin Sports Med.

[8] Kim SJ, Kee YM, Park DH, Ko YI, Lee BG (2018). Evaluation of the acromioclavicular joint morphology for minimizing subacromial erosion after surgical fixation of the joint using a clavicular hook plate. Clin Shoulder Elb.

[9] Helleberg F, Sobecki P, Józwiak R, Szaro P (2022). Anatomical variants of the acromioclavicular joint influence its visibility in the standard MRI protocol in patients aged 18-31 years. Surg Radiol Anat.

[10] Nakazawa M, Nimura A, Mochizuki T, Koizumi M, Sato T, Akita K (2016). The orientation and variation of the acromioclavicular ligament: an anatomic study. Am J Sports Med.

[11] Flores C, Celik H, Hoenecke H, D'Lima DD (2023). Subject-specific computational modeling of acromioclavicular and coracoclavicular ligaments. J Shoulder Elbow Surg.

[12] McLean A, Taylor F (2019). Classifications in brief: Bigliani classification of acromial morphology. Clin Orthop Relat Res.

[13] Nicholson GP, Goodman DA, Flatow EL, Bigliani LU (1996). The acromion: morphologic condition and age-related changes. A study of 420 scapulas. J Shoulder Elbow Surg.

[14] Viard B, Karp JS, Tremlet J, Asali Z, Trouilloud P, Trost O (2013). Unilateral duplication of the acromioclavicular joint: case report and literature review. Surg Radiol Anat.

[15] Cuomo F, Kummer FJ, Zuckerman JD, Lyon T, Blair B, Olsen T (1998). The influence of acromioclavicular joint morphology on rotator cuff tears. J Shoulder Elbow Surg.

[16] Beitzel K, Obopilwe E, Apostolakos J (2014). Rotational and translational stability of different methods for direct acromioclavicular ligament repair in anatomic acromioclavicular joint reconstruction. Am J Sports Med.

[17] Scillia AJ, Cain EL Jr (2015). Acromioclavicular joint reconstruction. Arthrosc Tech.

[18] Vereb M, Liepe K, Fischer M, Kaliska L, Noskovicova L, Balogova S (2018). Radiosynoviorthesis of acromioclavicular joint using 169Er-citrate: prospective evaluation of efficacy. Nucl Med Rev Cent East Eur.

[19] Polisetty T, Cannon D, Grewal G, Vakharia R, Levy JC (2023). Radiographic and anatomic variations on postoperative acromion fractures after inlay and lateralized reverse shoulder arthroplasty. J Shoulder Elbow Surg.

